# Supplementary material for: Stochastic processes constrain the within and between host evolution of influenza virus
Source: eLife. 2018 May 3;7:e35962. doi: 10.7554/eLife.35962 (PMC5933925; doi:10.7554/eLife.35962)
Supplement: Supplementary file 1. [file elife-35962-supp1.docx]

**Supplementary File 1. Sensitivity and s­pecificity of variant detection**

| Copy | Variant | Original Pipeline^b^ | | Current Pipeline^c^ | |
| --- | --- | --- | --- | --- | --- |
| Number^a^ | Frequency | Sensitivity | Specificity | Sensitivity | Specificity |
|  |  |  |  |  |  |
| >10^5^ | 0.05 | 1 | >0.9999 | 0.85 | 1.000 |
|  | 0.02 | 0.85 | 0.9999 | 0.15 | 1.000 |
|  | 0.01 | 0.95 | 0.9995 | - | - |
|  | 0.005 | 0.35 | 0.9999 | - | - |
|  |  |  |  |  |  |
| 10^4^-10^5^ | 0.05 | 0.95 | 0.9999 | 0.85 | 1.000 |
|  | 0.02 | 0.9 | 0.9999 | 0.15 | 1.000 |
|  | 0.01 | 0.8 | 0.9998 | - | - |
|  | 0.005 | 0.4 | 0.9999 | - | - |
|  |  |  |  |  |  |
| 10^3^-10^4^ | 0.05 | 0.8 | >0.9999 | 0.70 | 1.000 |
|  | 0.02 | 0.45 | 0.9999 | 0.15 | 1.000 |
|  | 0.01 | 0.2 | 0.9997 | - | - |
|  | 0.005 | 0.1 | 0.9999 | - | - |

^a^ Per µl transport media

^b^ As described in McCrone JT and Lauring AS, J. Virol. 90(15):6884, 2016.

^c^ As described in Methods, benchmarked for frequencies 0.02-0.98 only
