# Supplementary material for: Stochastic processes constrain the within and between host evolution of influenza virus
Source: eLife. 2018 May 3;7:e35962. doi: 10.7554/eLife.35962 (PMC5933925; doi:10.7554/eLife.35962)
Supplement: Supplementary file 2. [file elife-35962-supp2.docx]

**Supplementary File 2. Nonsynonymous substitutions in HA antigenic sites**

| House ID | | Enrolment ID | | Symptom Onset | | Subtype | | Frequency | Amino Acid Change | | Antigenic Site | | Vaccinated | | Day of Symptoms |
| --- | --- | --- | --- | --- | --- | --- | --- | --- | --- | --- | --- | --- | --- | --- | --- |
| 1111 | | 300481 | | 2011-03-30 | | H3N2 | | 0.071 | E62G | | E* | | No | | 0 |
| 1302 | | 301355 | | 2011-03-20 | | H3N2 | | 0.088 | L86I | | E | | Yes | | 1 |
| 2166 | | 320661 | | 2012-02-13 | | H3N2 | | 0.071 | V297A | | C | | Yes | | 1 |
| 3075 | | 331045 | | 2012-12-10 | | H3N2 | | 0.066 | I214T | | D | | Yes | | 1 |
| 5033 | | 50141 | | 2014-12-03 | | H3N2 | | 0.032 | A163T | | B | | Yes | | 2 |
| 5033 | | 50141 | | 2014-12-03 | | H3N2 | | 0.025 | D53E | | C* | | Yes | | 1 |
| 5033 | | 50141 | | 2014-12-03 | | H3N2 | | 0.023 | S312G | | C | | Yes | | 1 |
| 5034 | | 50143 | | 2015-01-11 | | H3N2 | | 0.119 | I307R | | C | | Yes | | 1 |
| 5034 | | 50143 | | 2015-01-11 | | H3N2 | | 0.161 | I307R | | C | | Yes | | 2 |
| 5147 | | 50630 | | 2014-11-18 | | H3N2 | | 0.164 | I242L | | D | | Yes | | 1 |
| 5219 | | 50935 | | 2014-12-05 | | H3N2 | | 0.175 | F193S | | B*^†^ | | No | | 3 |
| 5263 | | 51106 | | 2014-12-06 | | H3N2 | | 0.111 | T128A | | B | | Yes | | 3 |
| 5269 | | 51132 | | 2014-12-06 | | H3N2 | | 0.028 | I242T | | D | | Yes | | 2 |
| 5289 | | 51220 | | 2014-12-13 | | H3N2 | | 0.038 | K189N | | B*^†^ | | Yes | | -1 |
| 5302 | | 51273 | | 2014-12-13 | | H3N2 | | 0.03 | S262N | | E* | | Yes | | 0 |
| 4185 | | UM40738 | | 2013-12-14 | | H1N1 | | 0.021 | R208K | | Ca | | No | | 2 |
|  |  |  | |  | |  |  |  |  | |  | |  |  |  |

* Sites observed to vary between antigenically distinct strains in Wiley et al., 1981 and Smith DJ et al. 2004.

^†^  Sites that lie within the “antigenic ridge” described in Koel et al., 2013
