# Supplementary material for: Stochastic processes constrain the within and between host evolution of influenza virus
Source: eLife. 2018 May 3;7:e35962. doi: 10.7554/eLife.35962 (PMC5933925; doi:10.7554/eLife.35962)
Supplement: Supplementary file 3. [file elife-35962-supp3.docx]

Supplementary File 3. Estimated bottleneck size for individual transmission pairs

| Subtype | Donor sample collection | Recipient sample collection | Estimated transmission date^a^ | Donor age (years) | Recipient age (years) | Minority iSNV^b^ | Trans-mitted iSNV | Bottle-neck^c^ | 95% CI^d^ |
| --- | --- | --- | --- | --- | --- | --- | --- | --- | --- |
| A/H1N1 | 2011-02-23 | 2011-02-23 | 2011-02-21 | 7 | 40 | 2 | 1 | 4 | 2-11 |
| A/H3N2 | 2014-12-15 | 2014-12-15 | 2014-12-14 | 10 | 8 | 1 | 1 | 4 | 2-13 |
| A/H1N1 | 2011-02-23 | 2011-02-23 | 2011-02-21 | 7 | 4 | 2 | 1 | 3 | 2-10 |
| A/H3N2 | 2014-11-22 | 2014-11-25 | 2014-11-23 | 7 | 11 | 1 | 1 | 2 | 2-5 |
| A/H3N2 | 2014-11-25 | 2014-11-25 | 2014-11-24 | 11 | 9 | 1 | 1 | 2 | 2-3 |
| A/H3N2 | 2011-03-11 | 2011-03-11 | 2011-03-07 | 4 | 6 | 3 | 0 | 1 | 1-4 |
| A/H3N2 | 2011-03-02 | 2011-03-02 | 2011-02-28 | 9 | 7 | 1 | 0 | 1 | 1-22 |
| A/H3N2 | 2012-03-08 | 2012-03-12 | 2012-03-10 | 8 | 43 | 1 | 1 | 1 | 1-1 |
| A/H3N2 | 2013-01-04 | 2013-01-07 | 2013-01-01 | 43 | 5 | 4 | 1 | 1 | 1-2 |
| A/H3N2 | 2013-01-07 | 2013-01-04 | 2013-01-01 | 5 | 43 | 5 | 0 | 1 | 1-48 |
| A/H1N1 | 2014-01-07 | 2014-01-07 | 2014-01-03 | 1 | 6 | 7 | 0 | 1 | 1-9 |
| A/H1N1 | 2014-01-03 | 2014-01-07 | 2014-01-02 | 36 | 1 | 2 | 0 | 1 | 1-200 |
| A/H1N1 | 2013-11-27 | 2013-12-02 | 2013-11-27 | 2 | 5 | 1 | 1 | 1 | 1-4 |
| A/H1N1 | 2013-12-30 | 2013-12-30 | 2013-12-28 | 5 | 36 | 1 | 0 | 1 | 1-200 |
| A/H3N2 | 2014-12-25 | 2014-12-31 | 2014-12-24 | 37 | 6 | 2 | 0 | 1 | 1-40 |
| A/H3N2 | 2014-12-31 | 2014-12-25 | 2014-12-24 | 6 | 37 | 2 | 0 | 1 | 1-30 |
| A/H3N2 | 2014-12-25 | 2014-12-25 | 2014-12-24 | 37 | 4 | 2 | 0 | 1 | 1-29 |
| A/H3N2 | 2014-12-25 | 2014-12-25 | 2014-12-24 | 4 | 37 | 1 | 0 | 1 | 1-200 |
| A/H3N2 | 2014-12-31 | 2014-12-25 | 2014-12-24 | 6 | 4 | 2 | 0 | 1 | 1-39 |
| A/H3N2 | 2014-12-25 | 2014-12-31 | 2014-12-24 | 4 | 6 | 1 | 0 | 1 | 1-200 |
| A/H3N2 | 2014-12-08 | 2014-12-07 | 2014-12-06 | 5 | 7 | 1 | 0 | 1 | 1-200 |
| A/H3N2 | 2014-11-30 | 2014-12-07 | 2014-12-04 | 5 | 9 | 1 | 0 | 1 | 1-200 |
| A/H3N2 | 2014-11-26 | 2014-11-30 | 2014-11-29 | 8 | 38 | 1 | 0 | 1 | 1-200 |
| A/H3N2 | 2014-11-23 | 2014-11-25 | 2014-11-24 | 8 | 8 | 3 | 0 | 1 | 1-200 |
| A/H3N2 | 2014-12-09 | 2014-12-15 | 2014-12-10 | 15 | 6 | 1 | 0 | 1 | 1-200 |
| A/H3N2 | 2014-12-21 | 2014-12-24 | 2014-12-23 | 9 | 15 | 1 | 0 | 1 | 1-200 |
| A/H3N2 | 2014-12-21 | 2014-12-24 | 2014-12-23 | 9 | 11 | 1 | 0 | 1 | 1-200 |
| A/H3N2 | 2014-12-06 | 2014-12-09 | 2014-12-05 | 11 | 12 | 1 | 0 | 1 | 1-200 |
| A/H3N2 | 2014-11-17 | 2014-11-19 | 2014-11-17 | 9 | 36 | 4 | 0 | 1 | 1-200 |
| A/H3N2 | 2014-11-16 | 2014-11-17 | 2014-11-16 | 6 | 9 | 3 | 0 | 1 | 1-200 |
| A/H3N2 | 2014-11-10 | 2014-11-13 | 2014-11-12 | 40 | 2 | 1 | 0 | 1 | 1-200 |
| A/H3N2 | 2014-12-16 | 2014-12-15 | 2014-12-14 | 44 | 43 | 1 | 0 | 1 | 1-200 |
| A/H3N2 | 2014-12-12 | 2014-12-16 | 2014-12-13 | 11 | 44 | 1 | 0 | 1 | 1-200 |
| A/H3N2 | 2014-12-11 | 2014-12-16 | 2014-12-13 | 5 | 38 | 1 | 0 | 1 | 1-200 |
| A/H3N2 | 2014-11-30 | 2014-12-05 | 2014-12-03 | 6 | 40 | 3 | 0 | 1 | 1-22 |
| A/H3N2 | 2014-12-02 | 2014-12-04 | 2014-12-03 | 18 | 52 | 2 | 0 | 1 | 1-200 |
| A/H3N2 | 2015-01-06 | 2015-01-09 | 2015-01-04 | 6 | 6 | 5 | 0 | 1 | 1-37 |
| A/H3N2 | 2014-12-15 | 2014-12-15 | 2014-12-11 | 34 | 3 | 9 | 1 | 1 | 1-1 |
| A/H3N2 | 2014-12-20 | 2014-12-22 | 2014-12-21 | 10 | 36 | 1 | 0 | 1 | 1-200 |
| A/H3N2 | 2014-12-20 | 2014-12-22 | 2014-12-21 | 10 | 8 | 1 | 0 | 1 | 1-200 |
| A/H3N2 | 2014-12-26 | 2014-12-27 | 2014-12-26 | 47 | 46 | 2 | 0 | 1 | 1-14 |
| A/H3N2 | 2014-12-12 | 2014-12-18 | 2014-12-16 | 9 | 43 | 2 | 1 | 1 | 1-2 |
| A/H3N2 | 2014-12-22 | 2014-12-21 | 2014-12-19 | 9 | 4 | 2 | 2 | >200^e^ | NA |
| ^a^ Transmission was estimated to have occurred one day before symptom onset in the recipient host | | | | | | | | |  |
| ^b^ Alleles present between 2-50% in the donor host | | | |  |  |  |  |  |  |
| ^c^ Bottleneck estimated using the beta binomial model  ^d^ 200 represents the maximum bottleneck size tested | | | |  |  |  |  |  |  |
| ^e^ Both minority iSNV were found in both the donor and the recipient at nearly identical frequencies. | | | | | | | |  |  |
